# Barriers to rabies control through mass dog vaccination in rural Uganda: Insights from community perspectives and key informant interviews

**DOI:** 10.1371/journal.pntd.0013578

**Published:** 2025-10-09

**Authors:** Dickson Akankwatsa, Arthur Bagonza, Juliet Kiguli, Andrew Kambugu, Mohammed Lamorde, Samuel George Okech, Clovice Kankya, Doreen Agado, Felister Apio, Terence Odoch, Anna Mary Kahunde, Sonja Hartnack, Monique Léchenne, Frederic Lohr, Adrian Herrera, Salome Dürr

**Affiliations:** 1 Department of Community Health and Behavioral Sciences, Makerere University College of Health Sciences, School of Public Health, Kampala, Uganda; 2 Department of Global Health Security, Infectious Diseases Institute, Makerere University, Kampala, Uganda; 3 Department of Veterinary Pharmacy, Clinical and Comparative Medicine, College of Veterinary Medicine, Animal Resources and Biosecurity, Makerere University, Kampala, Uganda; 4 Department of Biosecurity, Ecosystems and Veterinary Public Health, College of Veterinary Medicine, Animal Resources and Biosecurity, Makerere University, Kampala, Uganda; 5 Department of Production Kyegegwa District Local Government, Kyegegwa, Uganda; 6 Section of Epidemiology, Vetsuisse Faculty, University of Zurich, Zurich, Switzerland; 7 Clinical Department for Farm Animals and Food System Science Centre for Veterinary Systems Transformation and Sustainability, University of Veterinary Medicine Vienna, Vienna, Austria; 8 Swiss Tropical and Public Health Institute, Swiss Center for International Health, Allschwil, Switzerland; 9 Worldwide Veterinary Service, Cranborne, United Kingdom; 10 Veterinary Public Health Institute, Vetsuisse Faculty, University of Bern, Bern, Switzerland; University of Abuja Faculty of Veterinary Medicine, NIGERIA

## Abstract

**Background:**

Globally, rabies claims about 59,000 human lives annually, and 99% of human transmission is by dogs. The disease is entirely preventable through mass dog vaccination. Despite this, only an estimated 10% of dogs in Uganda are vaccinated, and the reasons for this low uptake are not fully understood. This study investigated barriers to mass dog vaccination for rabies control in Kyegegwa, a rural Ugandan district.

**Methodology:**

This qualitative study was conducted with eight key informants (KIs) and twelve focus groups (FGs) with participants from twelve randomly selected parishes. Audio recordings were transcribed in English and transcripts were managed using Atlas ti version 6.0 data management software. Thematic analysis was conducted to produce the results.

**Results:**

This study identified three major themes affecting mass dog vaccination in Kyegegwa District: (1) community dynamics, including misconceptions about vaccines, limited awareness, and difficulties in handling aggressive dogs; (2) Resource and service constraints such as vaccine shortages, limited veterinary personnel, and high costs; and (3) Systemic and operational challenges, including delayed campaign announcements, poor communication, and lack of vaccination certificates. These barriers collectively explain the persistently low coverage of mass dog vaccination in rural Uganda.

**Conclusion:**

Limited awareness, logistical challenges, and inadequate veterinary resources were reported to be barriers to rabies control in Kyegegwa District. Addressing these issues may require timely communication, workforce strengthening, animal mobile clinics, and cost mitigation. Accurate dog population assessments and tailored community strategies may improve vaccination coverage, offering a foundation for effective rabies prevention in Kyegegwa District and similar resource-limited settings.

## Author summary

Rabies, a fatal viral disease preventable through mass dog vaccination, remains a public health challenge in Uganda, where dog vaccination coverage is only 10%. This study investigated the barriers to mass dog vaccination campaigns in Kyegegwa, a rural Ugandan district. Using focus group discussions (FGDs) and key informant interviews (KIIs), we identified three critical themes: community dynamics, resource and service constraints, and systemic and operational challenges. Limited community awareness and misconceptions, such as belief that vaccinated dogs lose their ability to bark, hindered participation in vaccination campaigns. Additionally, challenges in managing aggressive dogs and the absence of accurate dog population data further impeded effective vaccination planning. Resource constraints restricted access and coverage, including vaccine shortages, insufficient veterinary personnel, and high costs. Operational barriers such as poor communication, delayed campaign announcements, and a lack of vaccination certification exacerbated the problem.

To address these challenges, strategies such as accurate dog population assessments, mobile vaccination units, community-centered sensitization campaigns, and financial subsidies for low-income dog owners are crucial. Strengthening veterinary infrastructure and enhancing timely communication can significantly improve vaccination efforts. These findings suggest the importance of tailored interventions and community engagement to achieve the global goal of eliminating dog-mediated human rabies deaths by 2030, particularly in resource-limited settings like rural Uganda.

## Introduction

Rabies is a preventable viral zoonotic disease that causes acute encephalitis and is almost consistently fatal once symptoms appear [1]. The disease continues to pose a public health challenge, particularly in developing regions of Africa and Asia. Globally, rabies is responsible for an estimated 59,000 human deaths annually, with domestic dogs accounting for 99% of human cases [1,2]. Children under 15 years old are disproportionately affected, representing approximately 40% of rabies deaths [3]. Despite being preventable through dog vaccination and post-exposure prophylaxis (PEP), dog-mediated human rabies persists in many parts of the world due to poverty, inadequate healthcare infrastructure, and poor disease awareness [4].

The global initiative “Zero by 30” led by the World Health Organization (WHO) and its partners, aims at eliminating dog-mediated human rabies deaths by 2030 [5,6]. This strategy emphasizes achieving a 70% vaccination coverage among dogs, enhancing public awareness, and ensuring timely access to PEP for people after being bitten by a potentially rabid animal. Evidence has shown that vaccinating 70% of the canine population effectively interrupts rabies transmission [7]. However, attaining this target has proven challenging in Sub-Saharan Africa due to factors such as limited vaccine availability, logistical constraints, socio-economic challenges, and inadequate community engagement [8].

Drawing on evidence primarily from sub-Saharan Africa, numerous studies have explored the barriers and facilitators influencing effective mass dog vaccination campaigns. Key barriers identified include insufficient public information, unavailability of dogs and their owners during vaccination efforts, challenges in handling dogs, ineffective monitoring systems, limited funding, and the influence of geographic, cultural, and religious diversity [9–11]. Across these settings, four structural features explain why these barriers persist. First and foremost, there has been progressive privatization and decentralization of veterinary services since the 1990s, which has shifted costs to dog owners [12]. Secondly, long-standing deficits in rural infrastructure and cold-chain capacity steadily reduced the reach of dog vaccination campaigns [13]. In addition, fragmented One Health governance limits coordination between veterinary and human health departments [14]. Finally, there is intense competition for limited district resources amid multiple concurrent public health priorities.

Some studies have highlighted the importance of participatory approaches that involve communities and local authorities, emphasizing the need for effective communication channels and locally adapted vaccination strategies, such as static point and door-to-door methods [9]. For instance, in Tanzania’s Kilombero and Ulanga districts, poor campaign mobilization, inappropriate timing, unsuitable static point locations, and inadequate equipment and staffing were found to affect community compliance with dog vaccination campaigns [15]. Similarly, research in Burkina Faso revealed that factors such as local dog breeds, low income, and limited education levels negatively influenced dog owner participation, while providing cost-free vaccination and raising community awareness were shown to enhance participation [8,16]. Although interventions such as offering incentives, like dog collars, have been implemented to improve participation, vaccination coverage in many settings has remained below the WHO-recommended threshold of 70% [17]. In particular, the low turnout at static vaccination points often remains poorly understood, underscoring the need for further investigation into these barriers to inform more effective vaccination strategies.

Uganda faces similar challenges, with dog-mediated human rabies remaining endemic, particularly in rural areas where nearly 90% of the population resides [17,18]. A recent KAP survey conducted in Uganda shows that routine vaccination is not practiced in most Ugandan communities and that culling of suspected dogs is common practice [19]. Despite government efforts to provide rabies vaccines free of charge, dog vaccination coverage remains critically low, at an estimate of 10%, which is far below the recommended WHO threshold [5,20,21]. Household studies have linked the low coverage to poverty-related barriers and weak access to veterinary care [22]. Moreover, an analysis of One Health governance constraints in Uganda has shown that fragmented mandates and inadequate funding streams further impede vaccination campaigns [23]. While previous studies have identified barriers such as lack of awareness and logistical difficulties, there is limited understanding of the unique challenges faced in Uganda’s rural settings [22,24,25].

This study was conducted to address this knowledge gap and explore barriers to mass dog vaccination for rabies control in Kyegegwa District, in rural Uganda, using a qualitative approach. Qualitative research offers the capacity to delve into stakeholders’ own language, beliefs and decision processes that would not have been otherwise captured by quantitative methods [26]. This qualitative study aimed at exploring the perspectives of participants about barriers to mass dog vaccination. It was envisaged that the study results would inform targeted strategies to improve dog rabies vaccination coverage in Kyegegwa district, while offering insights relevant for other Ugandan districts and similar contexts in sub-Saharan Africa.

## Methods

The methods, analysis and results sections are written in accordance with the Consolidated Criteria for Reporting Qualitative Research (COREQ) guidelines [27].

### Ethics approval and consent to participate

Ethical clearance to conduct this study was granted by the Makerere University School of Public Health Research Ethics Committee (reference number, SPH-187). Additionally, the study protocol was approved by the Uganda National Council for Science and Technology (HS3463ES). Permission to conduct the research was also secured from the District Health Officer (DHO) of Kyegegwa district. Literate participants provided signed informed consent, while those unable to read and write were briefed orally by a bilingual research assistant who presented the information sheet in Runyoro-Rutooro (participants’ preferred local language). The research assistant explained the purpose, procedures, risks, and benefits, and answered any questions. The participants then gave consent using thumbprints before the study’s commencement. To ensure confidentiality, trust and streamline the transcription process, identification numbers were assigned to participants before the interviews began.

### Study area and setting

Kyegegwa District, located in the Midwestern region of Uganda, covers an area of 1,747 km^2^ (675 square miles). The district lies along the main highway connecting Mubende and Fort Portal Tourism City, approximately 110 kilometers (68 miles) east of Fort Portal. It shares borders with Mubende District to the West, Kiruhura District to the south, Kibaale District to the North, Kyenjojo District to the Northwest, and Kamwenge District to the southwest. The district headquarters are in Kyegegwa Town Council, which is 500 meters off the highway. Kyegegwa Town Council serves both the administrative and commercial hub of the district. Other notable trading centres include Mpara, Kakabara, Hapuuyo, Kazinga, and Kasule.

The study was conducted between July and October, 2023 in four sub-counties and twelve parishes within Kyegegwa district. These sub-counties and their respective parishes were: Nkanja (Bujubuli, Kakoni, Kyabirikuya), Kyatega (Katamba, Kyatega, Nkomangani), Ruyonza (Kisagazi, Karwenyi, Kijongobya), Mpara (Kisambya, Bugido and Mpara Central).

Kyegegwa District is predominantly rural, with an estimated population of approximately 501,120 residents as per the Uganda National Population and Housing Census of 2024 [28]. The local economy is primarily based on mixed farming and small-scale trading, and the community reflects diverse cultural and religious backgrounds, with Christianity being the predominant faith. There are no documented publicly available estimates of dog population in the district, however, according to the district veterinary office, Kyegegwa is home to approximately 25,000 dogs, averaging 300 dogs per parish and 25 dogs per village.

In terms of veterinary healthcare, Kyegegwa District has made strides in meeting the growing needs of its population, though some disparities still persist. The veterinary sector is headed by the district veterinary officer (DVO), who is deputised by the senior veterinary officer. Each of the sub-counties is meant to have one assistant animal husbandry officer who reports to the sub-county chief and the DVO. The assistant animal husbandry officers are directly supervised by the senior veterinary officer of the district. Currently, Ruyonza and Mpara Town Council do not have assistant animal husbandry officers due to resource gaps, and these are being caretaken by the senior veterinary officer.

### Study design

This study employed a qualitative-descriptive design to document community members’ subjective perceptions of rabies-vaccination campaigns while maintaining a low level of interpretive abstraction from researchers as has been done elsewhere [29,30]. We considered that the researchers’ role was that of neutral listeners who catalogued participants’ accounts and organised them thematically, rather than generating explanatory theory. This informed both data-collection (open-ended guides, non-directive facilitation) and rigour strategies (dual independent coding) and explains why we undertook validation measures such as triangulation (described later on in the methods) [31,32].

### Sampling and recruitment procedures

This qualitative study utilized key informant interviews (KIIs) and FGDs to gather data. Purposive, maximum-variation sampling was employed to capture a wide spectrum of community and professional voices involved in dog-rabies control in Kyegegwa district. Between 3^rd^ July and 31^st^ October 2023, adult dog-owners as well as the aforementioned stakeholders were invited from twelve randomly selected parishes to FGDs. Eligibility for FGDs was; being a resident in the parish for more than 12 months and direct responsibility for, or regular contact with, at least one dog. During purposive sampling, we aimed for maximum variation by gender, age group and role (dog-owner, community health worker, local council representative). Community leaders introduced the study, after which the research team approached participants face-to-face by a research assistant. Sampling and recruitment stopped when saturation—defined as the point at which no new information relevant to the research question arose in successive interviews, was achieved [33]. After every KII or FGD, two analysts independently compared emergent codes with the cumulative codebook after which discrepancies and potential new information were discussed with the senior advisor for qualitative research until consensus was reached. The study was planned in two waves. Wave 1 comprised five KIIs and eight FGDs. Weekly reviews showed that novel codes still appeared, so a second wave of three KIIs and four FGDs was conducted. In this study, code saturation (the point at which no new codes or themes emerged from successive transcripts) was unanimously reached by the 10^th^ FGD and the 6^th^ KII, although two pre-scheduled additional sessions in each stream were conducted to confirm redundancy and respect prior agreements with community members, after which data collection ceased for pragmatic and ethical reasons [34]. Letters of invitation were sent to KIs, followed by a telephone call to confirm participation. Focus group discussions were conducted with community members as recommended by focus-group methodologists, and are consistent with empirical work on saturation in documented qualitative studies [35,36]. Empirical work on FGD saturation suggests that, when the topic is narrowly bounded and participants share contextual knowledge, 8–12 participants per group provide breadth and depth of data [35]. The decision, therefore, balanced the specificity of the research question, the heterogeneity required within each group, and the pragmatic constraints of convening rural participants who travel long distances.

To improve rapport, the area local council chairpersons first introduced the study and endorsed the field team, after which facilitators engaged participants in informal conversation and confirmed their language [37]. All interviews were led by trained researchers who shared cultural and linguistic background with the study participants. Ice-breakers preceded topic questions, and facilitators emphasized voluntary participation. These relational practices built trust, while confidentiality was protected by assigning each participant a numeric code used in transcripts and reports. Focus group participants were approached face-to-face by the area’s local council chairperson under the guidance of the researchers. Sixteen invited individuals did not participate in the FGDs, with 11 reporting they missed the invitation and five citing other commitments on the scheduled dates.

### Data collection

Data collection was carried out by a team of researchers between 3^rd^ July, 2023 and 31^st^ October, 2023. One researcher (DA) conducted the KIIs, while FGDs were facilitated by three researchers (AMK, TO and DA). Interview guides were developed to gather information from participants through FGDs and KIIs. The interview guides for data collection were initially developed in English by the research team and then translated into the local dialects (Runyoro-Rutooro) to ensure effective communication with participants. These guides included prompts for ensuring comprehensive data collection and were extensively reviewed by the research team for relevance and clarity. Prior to data collection, as recommended by various scholars, the guides were piloted-tested in non-study parishes within Kyegegwa District to confirm their ability to elicit the intended information and adapted thereafter [38,39]. The guides, which included carefully designed prompts and were thoroughly reviewed by the research team, served as tools for collecting information from community members. Key questions posed to participants included: What are your overall thoughts about dog vaccination? What challenges do you think dog owners face that stop them from taking their dogs for vaccination? amongst others. Each question had multiple probes to guide the interviewer to obtain sufficient information regarding the barriers to dog mass vaccination as necessary. Information was deemed to be sufficient when participant responses addressed the core dimensions of the question, and no new details relevant to the research question emerged upon further prompting. No repeat interviews were conducted.

FGDs were conducted in Runyoro-Rutooro, the local language of participants, in locations where participants felt comfortable and offered a degree of privacy and minimal noise. Such locations included a school, parish hadquarters, under a tree, or in a church. KIIs were held in English, in the participants’ offices, to provide a convenient and quiet setting. Only the researchers and participants were present during the discussions and interviews, and no additional demographic information was collected about the participants. All FGDs and interviews were audio-recorded, and field notes were taken during interviews to supplement the recorded data. FGDs lasted between 30 and 60 minutes, while KIIs ranged from 10 to 30 minutes. Data saturation for FGDs was reached by the 10th session, and for KII by the 6th interview. To ensure no additional information emerged, two more FGDs and KIIs were conducted beyond the point of saturation.

We implemented several validation steps, in line with our assumption that the role of researcher(s) was to act as a neutral observer to collect and organise information on participants’ perspectives. First, an independent research assistant cross-checked each transcript against the audio recordings to correct any transcription errors. Second, field notes were systematically compared to transcripts during weekly debrief sessions, and any inconsistencies were discussed and resolved by the analysis team. Third, we triangulated findings across FGDs and KIIs, ensuring that emergent themes reflected multiple data sources. Finally, an audit trail recorded all coding decisions, code-book updates and analytic memos, providing transparency and supporting the credibility of our thematic analysis.

### Researcher characteristics

At the time of the study, DA was a Master’s student and a Global Health Security Program Officer. AMK holds an MSC and is a senior veterinary officer with Kyegegwa District Local Government, while TO has a PhD and is a senior Lecturer at Makerere University, Kampala, Uganda.

DA and TO are male, and AMK is female. DA had training in qualitative research methodology, while AMK and TO had prior experience in qualitative research. No prior relationship existed between the researchers and most participants, although some participants were familiar with AMK through her work in the district. None of the participants knew DA or TO, but they were informed that a team from Makerere University would be visiting for discussions on dog vaccination. DA expressed a specific interest in the research topic, while TO and AMK were part of a broader research project focused on rabies elimination.

### Data management and analysis

All audio recordings were transcribed verbatim into Runyoro-Rutooro, then translated into English by one of two bilingual research assistants (native Runyoro-Rutooro speakers with advanced training in translation). To ensure fidelity, a 10% random sample of translated transcripts underwent back translation by an independent assistant, and the senior qualitative researcher spot-checked key excerpts against the original audio. Any discrepancies in wording were discussed and resolved during weekly analysis debriefs, during which translators, coders and the study lead reconciled terminology and confirmed that conceptual meanings were retained. The verified transcripts were then uploaded into Atlas ti version 6.0 for coding and subsequent analysis. Two researchers (DA and TO) were responsible for coding the data. Initially, both researchers independently coded one FGD and one KII. Following this, a collaborative discussion was conducted to harmonise the codes, resolve any discrepancies, and develop a coding framework. This process enhanced the validity and reliability of the coding process. The remaining transcripts were then coded using the agreed-upon coding framework. Discussions and consensus-building were similarly applied during the identification of subcategories, categories, and themes to maintain the rigor of the analysis.

We conducted codebook thematic analysis (TA) [40] to generate a structured yet flexible catalogue of barriers and enablers to mass dog-rabies vaccination. Codebook TA was selected because the qualitative-descriptive design required; systematic coding that could be replicated by two analysts, the capacity to blend inductive insights with deductive categories informed by One Health literature, and an audit trail suitable for meaning-saturation monitoring.

Analysis followed a series of iterative phases, beginning with data familiarisation and moving to open, line-by-line coding of the initial seed transcripts. A provisional codebook was then developed by combining emergent themes with priori codes, after which all remaining transcripts were double-coded and any discrepancies resolved through consensus. Although we began with a short, descriptive code-book to guide double-coding, new codes were added inductively throughout analysis, and all themes were generated inductively from patterns in the fully coded data rather than imported from the initial code-book. Related codes were grouped into descriptive themes that summarised participant talk, and these themes were finally reviewed against the full dataset to ensure both coherence and distinctiveness.

Dual-coder consensus and an evolving codebook align with the coding-reliability features of codebook TA, while themes remained at the semantic level, reflecting participants’ expressed views rather than deeper latent meanings. Meaning saturation was declared when two consecutive transcripts fitted the existing codebook without extension FGD 10 and KII 7), after which no further codes or themes emerged [35]. Member checking of the themes was not undertaken because, in codebook TA, trustworthiness is established through transparent coding procedures and analyst triangulation rather than participant validation.

The code-book thematic analysis was operationalised through the qualitative content analysis steps proposed by Graneheim and Lundman [41]. Their framework guides the systematic abstraction of meaning units into condensed meaning, codes, categories and finally themes—an approach that aligns with the descriptive aim of this study which was to stay close to participants’ language while organising the material coherently. Manifest content was defined as the explicit, surface-level meaning of statements, whereas latent content referred to underlying patterns or ideas inferred across several passages. Coding prioritised manifest meaning; latent meaning was recorded only when at least two analysts agreed that multiple excerpts pointed to the same implicit idea.

A major theme had to appear in more than half of the FGD or KIIs as well as advance the understanding of barriers or enablers to dog-rabies vaccination as articulated by participants, even if mentioned less frequently. Themes, along with supporting data and quotations, are comprehensively presented in the results section. Quotations are presented, with participant codes and ID assigned to ensure anonymity and linked to the corresponding statements. The findings are reported clearly and consistently to accurately reflect the data collected.

## Results

### Participants of the FGD and KII

In total, the study involved eight KIs and twelve FGDs with 128 community members. The KIs were selected for their expertise and influence in rabies control and included the District Health Officer, District Veterinary Officer, Assistant District Health Officer in charge of the environment, Senior Administrator, District Inspector of Schools, a cultural leader, a religious leader, and the chairman of the local council five. Their professional experience in rabies-related work ranged from 3 to 17 years (median = 9 years).

Of the 128 FGD participants, 52% were women, with ages ranging from 18 to 67 years (median = 36). Most were subsistence farmers or petty traders, reflecting the rural economy of Kyegegwa District. A large proportion (82%) reported owning at least one dog, and all had previously heard of rabies. The groups were purposively selected to ensure variation by gender, age, and role (dog-owner, community health worker, local council representative), and were drawn from twelve parishes across four sub-counties.

### Outcomes of the FGDs and KIIs

The study findings are organized into three main themes: community dynamics and dog management, resource and service constraints, and systemic and operational challenges. The analysis first explores similarities and differences within FGDs, followed by those within KIIs, and compares variations across the two groups. Table 1 gives a summary of the key themes, sub-themes and representative quotes from the study participants.

**Table 1:** Summary of key themes, sub-themes and quotes.

Main theme	Sub-theme	Representative quote
Community dynamics and dog management	Limited awareness and misconceptions	“People here do not really know why dogs should be vaccinated. Some think it is only for big towns.” (P3 FGD Katamba) “And with vaccination, we have rumors around that if a dog is vaccinated, it no longer can bark, but otherwise I would find no problem with it.” (P6 FGD Kyabirikuya) “Lastly, the communities’ perception of rabies. People do not take it as a serious issue.” (KII 2)
	Dog management challenges	“My dog bites if a stranger even comes near. When the vet said I must bring him on market day I tried, but he snapped the rope twice and people ran away. Without a proper muzzle or cage I just gave up.” (P7 FGD Kyabirikuya)
	Dog population data gaps	“I don’t know how many dogs we have, so how can we know if enough are vaccinated?” (P5 FGD Bugido)
Resource and service constraints	Vaccine shortages and logistics	“Although the team vaccinated dogs last year, they only vaccinated dogs in the trading centres. The rest of the parishes were left out.” (KII 3)
	Inadequate veterinary workforce	“We have only one veterinary doctor who cannot reach all areas, especially during mass dog vaccination campaigns.” (KII 2)
	Cost of vaccination	“Privately, vaccines are available but costly, and without rabies cases to raise suspicion, it’s hard to get people to vaccinate their dogs.” (KII 3)
Systemic and operational challenges	Communication barriers	“We hear on the radio that the mass dog vaccination campaign is already happening; that is too late.” (P1 FGD Kyabirikuya)
	Misconceptions about vaccine effects	“Some people think vaccinated dogs become dull or run mad.” (P3 FGD Bujubuli)
	Lack of certification and follow-up	“People come to vaccinate their dogs, but certificates are not provided, and this demoralizes many from engaging.” (P2 FGD Kakoni)

### Community dynamics and dog management

Most FGD participants identified limited awareness as a barrier, pointing to widespread misconceptions, such as the belief that vaccinated dogs lose their ability to bark.

“*And with vaccination, we have rumors around that if a dog is vaccinated, it no longer can bark, but otherwise I would find no problem with it.*” (**P6 FGD Kyabirikuya**)

In addition, several participants emphasized that community members lacked information about dog vaccination and held misconceptions that discouraged participation.

“*People here do not really know why dogs should be vaccinated. Some think it is only for big towns.*” **(P3 FGD Katamba)**

Also, many participants stressed the importance of accurate dog population assessments to improve coverage, proposing a census approach in determining the exact number of dogs within communities. Frustration was evident over the lack of reliable data, which participants thought hindered planning and monitoring efforts.

“*I don’t know how many dogs we have, so how can we know if enough are vaccinated?*” **(P5 FGD Bugido)**

Additionally, FGD participants consistently highlighted challenges in managing and transporting aggressive dogs for vaccination, often making it difficult to ensure their inclusion in vaccination campaigns. For instance, several owners explained that even when they were willing to vaccinate, aggressive or nervous dogs were physically unmanageable during the long walk to the static point. One participant stated:

“*There are some dogs which are too aggressive, and it becomes difficult to take them for vaccination unless they vaccinate them from home.*” **(P6 FGD Kishagazi)**

Another participant said:

“*My dog bites if a stranger even comes near. When the vet said I must bring him on market day I tried, but he snapped the rope twice and people ran away. Without a proper muzzle or cage I just gave up.*” **(P7 FGD Kyabirikuya)**

Differences across FGDs emerged in the emphasis placed on certain challenges. For instance, while some groups focused on more aggressive dogs, others stressed logistical barriers, such as poor mobilization or late notice for vaccination campaigns, all of which led to a low turn-up, confusion and missed opportunities to participate in dog vaccination.

The majority of KIs talked about the widespread reluctance among community members to vaccinate their dogs, attributing this to a lack of perceived value in the vaccination process attributable to a lack of community sensitisation and public education.

Many informants felt that dog owners often do not see the importance of vaccinating their pets, viewing it as an unnecessary or low-priority activity. This reluctance was compounded by negative perceptions of both rabies and dog vaccination, with some community members underestimating the severity of rabies or dismissing the need for preventive measures.

“*The public doesn’t understand the importance of dog vaccination. There’s little information reaching them.*” **(KII 2)**

He added that:

“*Lastly, the communities’ perception of rabies. People do not take it as a serious issue.*” **(KII 2)**

KIs further emphasized the limited efforts to raise awareness and educate communities about the benefits of dog vaccination.

Differences within the KIs emerged in terms of what aspects of awareness were emphasized. While some informants referenced ongoing sensitisation efforts such as local radio or community meetings, others focused more on broader education and engagement gaps. This variation does not reflect opposing views, but rather differences in focus, suggesting that some aspects of rabies education may be more visible or salient to certain stakeholders depending on their roles.

Across the two groups, the authors noted similarities in the reported reluctance of dog owners, which they linked to widespread misconceptions and a lack of perceived value in vaccination. Based on participant responses, the authors interpreted a general lack of awareness, particularly around the importance and benefits of dog vaccinationas a major barrier reported in both FGDs and KIIs.

“*Another challenge is that some homes do not attach value on vaccinating these dogs…. and therefore people do not commit time to vaccinate their dogs.*” **(KII 1)**

On the contrary, differences were noted in the way FGDs placed a stronger focus on practical challenges such as the inability to manage aggressive dogs, roaming dog populations, and logistical barriers such as transporting dogs. KIIs on the other hand highlighted systemic challenges such as community mindsets, limited sensitization, and the need for policy interventions.

### Resource and service constraints

The majority of the participants across the FGDs consistently identified delays in vaccine availability and frequent shortages as challenges. They also highlighted the short duration of vaccination campaigns and the limited geographical reach of vaccination services as additional barriers to access. Scarcity of veterinarians was also mentioned as a pressing barrier, with participants expressing frustration over the long distances they needed to travel to reach vaccination sites and the insufficient number of veterinary personnel. Furthermore, many participants stressed that the high costs associated with vaccination deterred them from participating.

“*The hesitation to vaccinate dogs stems from the charge. People say they cannot afford to buy soap and salt, so how can they spend on dog vaccination?*” **(P3 FGD Kyabirikuya)**

There were variations among the different FGDs. While some groups emphasized logistical challenges, particularly the inaccessibility of vaccination sites, which was frequently cited as an obstacle for community members especially those residing in remote areas, other groups highlighted financial constraints as the most deterrent, emphasizing the prohibitive costs associated with vaccination as a key factor limiting participation.

The majority of KIs reported challenges related to vaccine shortages, particularly within public facilities, where demand often outstripped supply. These shortages were perceived as a major obstacle to achieving adequate vaccination coverage. Additionally, limited resources further compounded these challenges. While some regions had sponsored initiatives such as radio campaigns led by veterinary officers, these efforts were not widespread due to a lack of resources. This was evidenced from the DVO’s monthly activity log, which showed that in 2023, only 7 of 18 parishes received more than one static point clinic.

“*Although the team vaccinated dogs last year, they only vaccinated dogs in the trading centres. The rest of the parishes were left out.*” **(KII 3)**

Another KII said:

“*The mindset of dog owners is negative; they perceive pets as having no value.*” **(KII 5)**

Key informants also highlighted the lack of essential resources, such as vehicles required for vaccination outreach programs, which severely restricted the ability to deliver services to remote or underserved areas.

Another concern was the inadequate number of veterinarians available to support vaccination efforts. KIs noted that the few veterinarians in their communities often faced competing responsibilities, which limited their availability and capacity to provide timely and comprehensive vaccination services. This shortage of skilled personnel created gaps in service delivery and contributed to delays in effecting mass dog vaccinations.

“*We have only one veterinary doctor who cannot reach all areas, especially during mass dog vaccination campaigns.*” **(KII 2)**

Furthermore, most KIs raised concerns about the costs associated with vaccination documentation, such as certifications handed over to the dog owners upon vaccination. These costs were seen as an added burden, discouraging individuals from fully participating in vaccination programs. One KI said:

“*The vaccine is free, but the certification and preparation incur costs that vets need to recover.*” **(KII 1)**

Another KI intimated:

“*Privately, vaccines are available but costly, and without rabies cases to raise suspicion, it’s hard to get people to vaccinate their dogs.*” **(KII 3)**

Differences among KIs emerged in the degree of emphasis on private versus public vaccination services, with some KIs attributing delays to systemic challenges at the district and national level. One KI said:

“*Even if the vaccine is free, tying the dog and walking 10km is impossible for many dog owners.*” **(KII 1)**

In contrast, other key informants felt that low vaccination uptake was an attitudinal problem requiring stricter enforcement. For instance, one KI intimated that:

“*The core issue isn’t distance. It’s that community members don’t value vaccination, so we need stronger bylaws.*” **(KII 3)**

Recognising these divergent perspectives is crucial because interventions that address only logistical barriers, such as mobile clinics, may fail if officials simultaneously tighten enforcement without community buy-in.

Across FGDs and KIIs, vaccine shortages, inadequate veterinary services, and financial barriers were commonly reported challenges. Both agreed that these factors collectively hinder vaccination efforts. The responses from participants in the FGD focused on issues that could be regrouped as relating to logistical challenges and community-level frustrations, such as the limited duration of vaccination campaigns and the difficulty of accessing services in rural areas. This was a different orientation to the responses from KIIs, where participants focused on structural inadequacies, such as the lack of a dedicated vaccination budget and insufficient government support. The divergent emphases likely reflect the different roles and social contexts of FGD and KII participants—a subject we talk about in the discussion.

### Systemic and operational challenges

Delayed and inadequate communication regarding vaccination campaigns were reported from most FGDs. Participants frequently reported that the dissemination of information about upcoming campaigns was not timely, leaving many community members unprepared or unaware of when and where vaccinations would take place.

“*We hear on the radio that the mass dog vaccination campaign is already happening; that is too late.*” **(P1 FGD Kyabirikuya)**

Furthermore, the flow of information was described as inconsistent, with some participants expressing concern that they only learned about vaccination schedules through informal channels or after campaigns had already concluded. Misconceptions surrounding vaccination outcomes were also widespread barrier to participation. Some participants shared beliefs that vaccinated dogs might exhibit undesirable changes in behaviour, such as becoming dull or, paradoxically, rabid. These misconceptions not only fueled skepticism about the efficacy of vaccination but also discouraged broader community engagement. Additionally, participants expressed considerable frustration with operational inefficiencies in the vaccination process. A specific concern was the lack of certification or official documentation for vaccinated dogs. This absence of tangible proof was seen as undermining confidence in the system, as it left owners without verification of their compliance and, by extension, the credibility of the vaccination program itself. One participant stated:

“*People come to vaccinate their dogs, but certificates are not provided, and this demoralizes many from engaging.*” **(P2 FGD Kakoni)**

Another stated:

“*Some people think vaccinated dogs become dull or run mad.*” **(P3 FGD Bujubuli)**

Differences across FGDs were primarily in emphasis. While some groups highlighted communication failures, others focused more on misconception and the lack of operational follow-through.

The majority of the KIs observed that dog vaccination is often deprioritized compared to other livestock vaccinations, such as those for foot and mouth disease. They noted that this lower prioritization contributes to gaps in vaccination efforts for dogs. Similar to findings from FGDs, KIs reported prevalent misconceptions among dog owners, including the belief that vaccinated dogs might become rabid, which discourages participation. Additionally, KIs highlighted the lack of clear planning and advocacy by veterinary departments to emphasize the importance of dog vaccination, further limiting its implementation and impact.

“*Planners need to show the importance of vaccinating dogs to improve participation.*” **(KII 1)**

Another stated:

“*Vaccination of dogs is not a priority compared to cattle vaccination.*” **(KII 5)**

Differences among KIs arose in their focus on systemic barriers versus community-level challenges, with some attributing low turnout to lack of policy emphasis and others to misinformation.

Both KIIs and FGDs identified operational inefficiencies as key challenges, including communication delays and the lack of follow-up measures, such as issuing vaccination certificates. Misconceptions about the effects of vaccination were also prevalent, reported in both FGDs and KIIs. While FGDs emphasized community-level experiences, such as the frustration stemming from undelivered promised services, KIIs offered a broader systemic perspective. Key informants stressed the need for stronger policy-level prioritization and more effective advocacy by planners to address these issues.

## Discussion

This study explored the barriers to rabies control through mass dog vaccination in Kyegegwa District, a rural area in Uganda. The research was grounded in a larger research project seeking to eliminate rabies through electronic surveillance and a One Health approach, incorporating enhanced surveillance systems, integrated bite case management, and community engagement. Despite these efforts, challenges persist in reducing dog bites and controlling rabies-related morbidity and mortality. Three key themes emerged from the findings: community dynamics and dog management, resource and service constraints, and systemic and operational challenges. Together, these findings highlight practical gaps that hinder effective dog vaccination campaigns and provide specific insights for strengthening rabies prevention strategies in similar rural settings.

The findings of this study reveal that participants reported misconceptions about vaccination outcomes were widespread within their communities, including beliefs that vaccinated dogs could become rabid or lose their ability to bark, suggesting persistent gaps in public understanding of prevention. This reflects both individual and community level gaps in rabies awareness. Similar barriers have been noted in another low-resource setting in Tanzania, where locally held beliefs undermined the perceived value of vaccination [42]. Health systems may benefit from adopting culturally appropriate and locally tailored communication strategies to address misconceptions and enhance public knowledge about rabies prevention. Engaging communities in these efforts is essential as it encourages a sense of ownership, increases participation in vaccination campaigns, and builds trust in health interventions.

Participants also highlighted the importance of accurate dog population assessments as a foundational step for improving vaccination coverage. The inadequate data on dog populations emerged as a source of frustration, as it complicates planning and monitoring. This has also been reported in other studies. For example, in Sri Lanka, researchers emphasized the importance of precise dog population data for planning effective vaccination campaigns, using household surveys, vaccination points, and collar-marking to estimate dog populations and identify unvaccinated dogs [43]. These insights emphasize that data is not only crucial but foundational for efficient and targeted rabies vaccination efforts. Without it, resources may be wasted, or coverage may be inadequate in high-risk areas. Health systems may benefit from adopting standardised methods like household surveys, vaccination point records, and technologies such as GPS mapping or mobile applications to enhance data accuracy and support effective campaign planning.

Furthermore, participants consistently raised concerns about the challenges of managing and transporting aggressive dogs for vaccination. These challenges often hinder the inclusion of such dogs in campaigns, reducing overall coverage. These findings are in tandem with studies highlighting that some dogs develop behavioural changes, including aggression, after receiving the rabies vaccine [44,45]. These changes can make managing and transporting these dogs for future vaccinations difficult, potentially excluding them from vaccination campaigns. One potential solution could be to implement mobile clinics or specialised vehicles to address logistical challenges in transporting aggressive dogs for vaccination. Educating dog owners and engaging communities can therefore create a more supportive environment for rabies control initiatives.

The study revealed several barriers to accessing rabies vaccination, including delays in vaccine availability, frequent shortages, short campaign durations, and limited geographical coverage of vaccination services. These findings are consistent with studies from other low-and lower-middle-income countries, where it is mentioned that delays in rabies vaccine distribution are a common challenge. Similarly, rural and pastoral regions often encounter additional obstacles, such as low dog population densities and restricted access to vaccination services, further hindering effective rabies control efforts [46]. To address vaccine delays and shortages, health systems must strengthen supply chain management through improved forecasting, procurement, and distribution. In addition, extending the length of campaigns or implementing rolling vaccination strategies could improve accessibility and participation.

This study identified scarcity of veterinarians, long travel distances to vaccination sites, and the high costs associated with vaccination as barriers to mass dog vaccination. These findings are consistent with studies conducted in other settings, which highlight a shortage of health infrastructure and veterinarians often result in inadequate coverage of vaccination campaigns [11,15].

Similarly, the distance to vaccination sites is a well-documented barrier, particularly in rural areas where dog owners frequently face long, time-consuming, and costly journeys to access these services [11,25]. Moreover, many dog owners in low-income communities struggle to afford the associated expenses, including transportation costs, further limiting their participation in vaccination efforts [21]. Investing in training, recruiting, and deploying veterinarians in underserved areas is essential to address workforce shortages. Mobile vaccination units could improve accessibility by reducing travel distances and associated costs for dog owners. Additionally, providing financial subsidies or free vaccination services can alleviate cost barriers for low-income communities, boosting participation and coverage. Some proposed strategies, such as bundling rabies vaccination with existing community health days such as child immunisation or market outreach, may offer cost-efficient co-benefits. These integrated models could improve feasibility in low-resource settings by leveraging existing infrastructure while reducing incremental costs.

The contrast between the largely logistical concerns voiced in FGDs and the systemic constraints highlighted in KIIs is unsurprising when one considers both participant roles and data collection context. End users speak from immediate experience and, in a group, frustration about distance, cost, and handling difficult dogs is reinforced through shared storytelling [47,48]. By contrast, district officials working at policy level are primed to view the same problem through a lens of fragmented budgets and unclear mandates. Methodologically, this triangulation underlines why multi-level sampling matters. Had we relied on FGDs alone, governance barriers would have been under-represented. Had we relied on KIIs alone, voices about everyday access hurdles would have been minimized. It is therefore important that intervention designs pair community-led solutions such as mobile clinics with policy-level reforms such as earmarked rabies funds, reflecting insights from both data streams.

Delayed and inadequate communication about vaccination campaigns emerged among challenges. Information dissemination about upcoming campaigns was often untimely, leaving many community members unprepared or unaware of when and where vaccinations would occur. These findings align with studies on COVID-19 vaccination communication, which revealed similar shortcomings [49]. In those campaigns, the lack of timely, updated information delivered through adaptable channels left audiences without essential guidance. Moreover, reliance on information dumping rather than strategic, audience-focused communication overwhelmed audiences and obscured accurate, credible messages.

Health systems may benefit from prioritizing the timely dissemination of clear and actionable information about vaccination campaigns. Delays in communication undermine preparedness, reduce participation, and hinder overall coverage and impact. Utilizing diverse and adaptable channels like social media, radio, and community-based networks can enhance reach and engagement, particularly in underserved or remote areas.

The study findings contribute to the literature on rabies control by demonstrating how structural, community, and operational factors intersect to influence vaccination uptake. In so doing, the study refines existing models of rabies elimination by integrating community voices that are often not captured in policy and programmatic debates.

Beyond theory, the study provides actionable entry points for intervention, including the need for accurate dog population assessments, mobile vaccination units, and locally tailored communication strategies. This context-specific information offers practical guidance for designing scalable approaches, not only for Uganda but also for similar resource-limited settings across sub-Saharan Africa.

### Study strengths and limitations

This study has provided current literature on the barriers to rabies control in a rural area of Kyegegwa District in Uganda. A key strength of the study was the choice of the type of study which allowed us to capture experiences from community and from people regarded as being more knowledgeable than the rest regarding mass dog vaccination. Secondly, triangulation was done by involving community members who are actively involved in keeping dogs and the more knowledgeable community members, which may have increased the validity and credibility of the study. However, the study faced limitations, including its single-district scope which only allows contextual use of the findings. Despite being limited to a single district, the study provides in-depth, localized insights into the barriers to rabies control, which can inform targeted interventions in similar rural settings.

### Future research

While this study identifies context-specific barriers and proposes potential strategies to improve dog vaccination coverage in rural Uganda, further research is needed to translate these recommendations into practice. Implementation trials, such as stepped-wedge or cluster-randomised designs may benefit from assessing whether bundling rabies vaccination with existing outreach for child immunization days or market-day events or deploying rolling mobile teams actually increases uptake, owner satisfaction, and cost-effectiveness. In addition, mixed-methods process evaluations may highlight operational challenges, community acceptability, and fidelity to intended protocols.

## Conclusion

This study identified barriers to rabies control in Kyegegwa District, including limited awareness, logistical challenges, and inadequate veterinary resources. Addressing these requires timely communication, workforce strengthening, mobile units, and cost mitigation. Accurate dog population assessments and tailored community strategies could improve vaccination coverage, offering a foundation for effective rabies prevention in resource-limited settings.

## Supporting information

S1 File Codebook from qualitative interviews on barriers to rabies control through mass dog vaccination in Uganda. Contains codes, subthemes, themes and anonymized illustrative quotations used in the thematic analysis.
(XLSX)
